# Cation-Chloride Cotransporters KCC2 and NKCC1 as Therapeutic Targets in Neurological and Neuropsychiatric Disorders

**DOI:** 10.3390/molecules28031344

**Published:** 2023-01-31

**Authors:** Patricia Lam, Julia Newland, Richard L. M. Faull, Andrea Kwakowsky

**Affiliations:** 1 Centre for Brain Research, Department of Anatomy and Medical Imaging, Faculty of Medical and Health Sciences, University of Auckland, Auckland 1023, New Zealand; 2 Pharmacology and Therapeutics, School of Medicine, Galway Neuroscience Centre, University of Galway, H91 W5P7 Galway, Ireland

**Keywords:** GABA, KCC2, NKCC1, Huntington’s disease, Alzheimer’s disease, Parkinson’s disease, schizophrenia, Down syndrome, epilepsy, neuropsychiatric, neurodevelopment

## Abstract

Neurological diseases including Alzheimer’s, Huntington’s disease, Parkinson’s disease, Down syndrome and epilepsy, and neuropsychiatric disorders such as schizophrenia, are conditions that affect not only individuals but societies on a global scale. Current therapies offer a means for small symptomatic relief, but recently there has been increasing demand for therapeutic alternatives. The γ-aminobutyric acid (GABA)ergic signaling system has been investigated for developing new therapies as it has been noted that any dysfunction or changes to this system can contribute to disease progression. Expression of the K-Cl-2 (KCC2) and N-K-C1-1 (NKCC1) cation-chloride cotransporters (CCCs) has recently been linked to the disruption of GABAergic activity by affecting the polarity of GABA_A_ receptor signaling. KCC2 and NKCC1 play a part in multiple neurological and neuropsychiatric disorders, making them a target of interest for potential therapies. This review explores current research suggesting the pathophysiological role and therapeutic importance of KCC2 and NKCC1 in neuropsychiatric and neurological disorders.

## 1. Introduction

Neurological disorders including Alzheimer’s disease (AD), Huntington’s disease (HD), Parkinson’s disease (PD), Down syndrome (DS) and epilepsy, and neuropsychiatric disorders such as schizophrenia and autism spectrum disorders (ASDs), have a severe impact on the affected individual. These diseases affect not only the individual but also have social and economic impacts. Recently, a potential link regarding the underlying mechanisms between such diseases has been investigated [1,2].

AD is a neurogenerative disease that impacts millions of people on a global scale. Individuals with AD have a progressive degenerative reduction in synaptic and neuronal density in the hippocampus which leads to cognitive, memory, and emotional regulatory deficits [1]. Anatomically, the hippocampus and the entorhinal cortex are the first brain regions to display damage [1]. Loss of excitatory cholinergic neurons is recognized as one of the best markers for AD, however it has recently become apparent that there are also alterations in numerous other neurotransmitter (NT) systems, such as the γ-aminobutyric acid (GABA)ergic system. While previous studies have observed that GABAergic neurons are quite resistant in AD, on the other hand, it has also been shown that the involvement of GABAergic terminals lead to atrophy and might progress AD [1,3,4,5,6]. In the cortex, somatostatin-positive neurons that co-express GABA are reduced in AD, supporting the notion that GABAergic dysfunction contributes to AD pathology [1].

HD is another neurodegenerative disorder affected by GABAergic dysfunction and is characterized by chorea, or involuntary movements [7]. As for AD, HD patients experience cognitive and mood disorders, and studies suggest a reduced inhibitory drive and enhanced neuronal excitation in HD may contribute to learning and memory deficits [7,8]. GABAergic dysfunction in HD has been explored previously and continues to be a topic of interest in terms of discovering novel therapies [9]. Individuals with DS, a trisomy 21 condition, also exhibit AD-like pathology, and the conversion of GABA activity from inhibitory to excitatory can lead to learning and memory deficits [10,11,12]. A study found a positive shift in reversal potential for GABA (E_GABA_) in a DS mouse model and excitatory GABA activity [11]. Previous studies found cognitive deficits in DS can be restored by using a GABA α5-selective inverse agonist, thus, indicating altered GABA signaling contributes to the disorder [13,14]. For PD, aberrant giant GABAergic currents were discovered in mouse models of the disease, suggesting alterations in striatal GABAergic systems in PD [15,16]. Thus, add-on treatments targeting GABA activity to current therapies have been suggested [17]. Epilepsy is another neurological disorder that is affected by alterations of the GABA system. GABA-mediated excitation has been reported in the subiculum of epileptic patients, strongly suggesting GABAergic dysfunction [18].

Schizophrenia, a neuropsychiatric disorder, also has abnormalities in the GABAergic system where the ratios of three genes related to GABA signaling are increased: *GAD1* (GAD67 and GAD25), *SLC12A2* (NKCC1), and *SLC12A5* (KCC2) in the prefrontal cortex [19,20]. Due to these genes being linked to the r s3749034 genotype which is a risk-associated SNP for schizophrenia, these abnormalities in GABA signaling are critical to brain development and may contribute to genetic risk for schizophrenia [19].

GABAergic dysfunction has been implicated in various diseases as mentioned above. Fragile X (FRX) and Rhett syndrome (RTT) are some of the ASDs affected by disruptions in GABA signaling. FRX is an inheritable form of intellectual disability and autism, where enhanced or reduced GABAergic post-synaptic currents (PSCs) have been reported [21]. At birth, FRX patients have high Cl^−^ in hippocampal neurons which lasts until adulthood. In RTT, a neurodevelopmental disorder classified under ASDs, it is suggested that GABAergic neurons deficient in methyl-CpG-binding protein 2 (MeCP2) become dysfunctional and contribute to RTT phenotypes [20,22]. 

The GABAergic system has the crucial role of maintaining neuronal excitability by acting as the main inhibitory NT of the brain, controlling multiple physiological and biochemical processes. Currently, the role that GABA plays in the maintenance and balance of excitation and inhibition (E/I) is a particular topic of interest [1,23]. The GABAergic system is involved in regulating cognition, memory and learning, motor function, circadian rhythms, neural development, adult and embryonic neurogenesis and sexual maturation [24]. The effect of GABAergic dysfunction could be critical to the progression of neurodegenerative diseases. Recently, it has been observed that GABA signaling plays a notable role in diseases such as AD, HD, and DS, where the dysfunction of this system affects the progression of these diseases [8,11,23]. 

The potassium-chloride cotransporter 2 (KCC2) and sodium-potassium-chloride cotransporter 1 (NKCC1) cation-chloride cotransporters (CCCs) are among the more recent targets of interest for intervention in neurodegenerative disease. Ideally, these targets might help mitigate symptoms of neurological diseases such as HD, AD, and others with similar underlying disease mechanisms affecting the E/I balance. These membrane proteins are responsible for regulating the movement of sodium (Na^+^), chloride (Cl^−^), and potassium (K^+^) across the plasma membrane. The movement of ions is achieved by combining transmembrane gradients of Na^+^, K^+^ and Cl^−^ [24]. The main function of GABA in the brain is synaptic inhibition that acts via Cl^−^-permeable GABA_A_ receptors. The precise regulation of the two CCCs—KCC2 and NKCC1—dictates the polarity of GABA_A_ receptor signaling [8,25]. In early development, NKCC1 is preferentially expressed over KCC2, resulting in the influx of Cl^−^ into the neuron and excitatory GABA activity. On the other hand, when KCC2 mediates Cl^−^ extrusion in mature neurons, it leads to inhibitory GABA activity. When the expression of NKCC1 or KCC2 is disrupted, the neuronal Cl^−^ gradient can collapse. This can result in the disruption of inhibition and contribute to numerous neurological and neuropsychiatric disorders [8,20].

This review provides an overview of studies regarding the contribution of KCC2 and NKCC1 in neurological and neuropsychiatric disease pathogenesis. While there are several disorders where alterations in CCCs are implicated, there is limited evidence to confirm this. Therefore, this review will focus on HD, AD, DS, epilepsy, schizophrenia, and autism. By understanding NKCC1 and KCC2 expression and functional alterations in human diseased brain tissue, and in animal models of these disorders, there is a new avenue for finding novel therapeutic options for these diseases to mitigate symptoms and improve human quality of life. 

## 2. The GABAergic Neurotransmitter System 

GABA is the primary inhibitory NT in intrinsic neurons of the brain where it acts to regulate excitatory neurotransmission [26,27]. GABA is synthesized by the enzyme glutamic acid decarboxylase (GAD), which exists as two major isoforms in the mammalian central nervous system (CNS): GAD65 and GAD67, which correspond to the 65 kDa and 67 kDa isoforms, respectively [28]. While GAD65 is identified as the isoform mainly localized to axon terminals and GAD67 is expressed in neuronal cell bodies and dendrites, most neurons express both isoforms [28,29]. Once GABA is synthesized, it is encapsulated in synaptic vesicles by the vesicular GABA transporter (VGAT) and released into the synapse from the axon terminal by a calcium-dependent exocytosis [30].

GABA is localized to inhibitory type 2 synapses which comprise 15% of all adult cortical synapses [26,31]. Most neocortical GABAergic neurons exist as local interneurons, but overall, account for 30% of nerve terminals within the central nervous system (CNS) [26]. GABA receptors which respond to GABA have different conformational, pharmacological, and functional properties, with subtypes within each receptor type defined by specific receptor subunit composition [32,33,34]. Of importance to the CNS are ionotropic GABA_A_ receptors (GABA_A_Rs) that mediate fast postsynaptic potentials in response to GABA, and metabotropic GABA_B_Rs which mediate slow responses [35]. GABA_A_Rs are gated Cl^−^ channels which permit a Cl^−^ influx into the cell in response to GABA. The influx of Cl^−^ hyperpolarizes the cell to reduce the likelihood of an action potential, thus, inhibiting neuronal activity. 

GABA is essential for information processing, synaptic function, synaptic communication, and excitatory regulation, among other roles. The fine regulation of neuronal excitation is important as the E/I balance is fundamental to a functioning CNS. The stimulation of a cell by glutamate, an excitatory NT, leads to an excitatory post-synaptic potential (EPSP). This is regulated by a fast GABA_A_-mediated inhibitory post-synaptic potential (IPSP) and a slow GABA_B_-mediated IPSP which follow shortly after, to prevent excessive neuronal excitation [36]. The polarity of GABA activity in the neuron, while influenced by many factors, is partially dictated by the relative expression of KCC2 and NKCC1 on the neuron. There is a period during neuronal development where GABA acts as an excitatory NT, which is worth considering when investigating disruptions to GABAergic signaling in the adult [25]. Depolarizing activity has also been reported during disease processes in the adult brain [25]. Hence, there is a need to focus on the influential factors surrounding GABA_A_R-mediated activity. 

## 3. Cation-Chloride Cotransporters in GABAergic Neurotransmission

### 3.1. KCC2 and NKCC1

CCCs cotransport Cl^−^ across cellular membranes to regulate cell volume. They are also involved in renal function, auditory function and modulate neuronal excitability [37]. KCC2 and NKCC1 belong to the solute carrier (SLC)12 family, which is composed of one Na-Cl, two Na-K-2Cl, four K-Cl cotransporters, and one homologous protein [38]. Their names are derived from the ions they transport across the cell membrane. NKCC1 is encoded by the *SLC12A2* gene and exists as a basolateral isoform. In the developing neuron NKCC1 is the primary Cl^−^ importer which cotransports 1 K^+^, 1 Na^+^, and 2 Cl^−^ into the neuron [37]. Meanwhile, KCC2 is encoded by the *SLC12A5* gene and, while lowly expressed in the immature neuron, becomes the predominant Cl^−^ exporter in mature neurons where it cotransports K^+^ and Cl^−^ in a 1:1 ratio [25,37]. 

### 3.2. KCC2 and NKCC1 and GABAergic Polarity

Synaptic inhibition mediated by the GABA_A_R needs high membrane conductance to prevent depolarization or excitation. The relative expression of KCC2 and NKCC1, depending on the maturity of the neuron, is important as it determines GABA signaling polarity. Ben-Ari et al. extensively explored this portion of GABAergic neuronal maturation whereby GABA signaling undergoes an excitatory-to-inhibitory switch in development. High expression of NKCC1 in immature neurons creates a less negative reversal potential for GABA (E_GABA_) by promoting Cl^−^ accumulation and, thus, excitatory GABA_A_ activity [25,39], whereas high expression of KCC2 in the mature neuron creates a more negative E_GABA_ by reducing the level of intracellular Cl^−^, and, thus, inhibitory GABA_A_ activity [40,41]. The excitatory-to-inhibitory switch during development is essential for the developing neuron [42]. The knock-out of NKCC1 in neurons results in delayed development of both GABAergic and glutamatergic synapses, indicating NKCC1 expression in immature neurons is required for the maturation of inhibitory and excitatory synapses [42]. The molecular mechanisms behind the excitatory-to-inhibitory switch in GABAergic neurotransmission involve serine 940 and threonine 906 and 1007, whereby phosphorylation of serine 940 promotes KCC2 activation, while phosphorylation of threonines 906 and 1007 on the intracellular C-terminal domain of KCC2 decreases its activity [43,44]. The with-no-lysine (K) kinase (WNK) is also involved in regulating KCC2 and NKCC1 phosphorylation, thus, indicating the critical role KCC2 and NKCC1 plays in the transition of GABA activity during development [45,46]. 

## 4. KCC2 and NKCC1 in Neurological Disorders

Numerous CNS diseases have indicated a disruption in GABA signaling, including HD, AD, DS, schizophrenia, and epilepsy [8,18,47,48]. It is plausible that altered expression of KCC2 and NKCC1 in such disorders contributes to detrimental GABA signaling and E/I imbalance. In the instance where NKCC1 is upregulated in the mature neuron, high intracellular Cl^−^ implies a reversion to immature physiology, thus, disrupting neuronal development, formation of synaptic connections and normal neuronal functioning [20]. 

### 4.1. Huntington’s Disease

Huntington’s disease (HD) is the first neurological disorder that shows evidence for the inhibitory-to-excitatory switch in GABA transmission [8]. HD is an autosomal dominant neurodegenerative disorder typically characterized by progressive motor incoordination and involuntary movements resulting from degeneration of the striatum and related neuronal pathways [7,8]. The cognitive and behavioral impairments involving the cortex and hippocampus are experienced early in the disease whereas the motor impairments are experienced more than a decade after. Individuals affected by HD display hippocampal-dependent learning and memory deficits [8]. HD mouse models have altered excitatory synaptic plasticity in the hippocampus as well as impaired spatial cognition. Even though hippocampal-dependent learning and memory do not rely specifically on the synaptic plasticity of glutamatergic synapses, it has been observed that inhibitory GABA-releasing interneurons are required for tasks requiring hippocampal-dependent learning and memory [8]. A previous study reported increased NKCC1 and decreased KCC2 expression in the hippocampus of HD mouse models [8]. This was notably accompanied by a depolarized E_GABA_, excitatory GABA_A_R signaling, loss of inhibitory drive, and enhanced neuronal excitation. Consequently, the R6/2 HD mice exhibited deficits in memory, and spatial and recognition learning tasks. As GABAergic inhibition is necessary for hippocampal-related learning and memory tasks, Dargaei and colleagues hypothesized that restoring GABAergic activity via bumetanide would restore learning and memory in HD mice, which was the case [8]. The reduced expression of KCC2 in R6/2 and YAC128 HD mice was accompanied by an increase in NKCC1, which together resulted in excitatory GABA in the hippocampi of HD mice. Importantly, NKCC1 inhibition by the U.S. Federal Drug Administration (FDA)-approved NKCC1 inhibitor, bumetanide, abolished the excitatory action of GABA and rescued the performance of R6/2 mice in hippocampal-associated behavioral tests. Furthermore, recent mouse proteomic studies revealed that the KCC2 encoding gene, *SLC12A5*, is highly enriched in the Htt proteome, but this interaction has not been validated yet [8]. Additionally, a bioinformatic analysis of the unfolded protein response (UPR)-regulated genes in HD found an increase in NKCC1 mRNA and a decrease in KCC2 mRNA. Impaired GABAergic signaling contributes directly to the impaired cognitive and motor functions that are seen in HD [8]. However, there are no studies that have examined the shift in KCC2 and NKCC1 expression in the human HD hippocampus. The investigation of KCC2 and NKCC1 expression presents a novel window into dissecting the disease pathology, as well as identifying a potential target to treat this disease [9,49,50].

### 4.2. Alzheimer’s Disease

AD is a neurodegenerative disorder that affects the aging population. AD patients typically present with memory loss and cognitive impairment due to the progressive loss of hippocampal neurons. The well-known hallmarks of AD pathology are the progressive accumulation of extracellular beta-amyloid (Aβ) plaques and intracellular neurofibrillary tangles (NFTs), which severely affect the hippocampus [23,51]. Consequently, there is substantial neuronal loss in the hippocampus and a functional deficit in learning and memory [51]. Cognitive function can decline with disruptions to neuronal network synchrony as working memory relies on the synchronized activity of excitatory and inhibitory networks [52,53,54]. It is apparent that AD pathology contributes to network dysfunction as hippocampal neurons demonstrate increased neuronal activity in AD [53,55,56,57,58].

The underlying mechanisms surrounding Aβ_1–42_-induced network instability are unclear but have historically been attributed to dysfunction in the glutamatergic and cholinergic NT systems [23]. Evidence in recent years has since revealed that the GABAergic system, which was previously thought to be unaffected, is also altered in AD [3,5,23,59]. Studies have shown that neuronal hyperactivity is attributed to reduced GABA activity instead of enhanced glutamatergic activity [3,5]. Hippocampal GABAergic interneurons are also selectively lost early in AD pathogenesis in animal models [6,60]. There is growing interest in targeting the GABAergic system, as treating apolipoprotein E4 knock-in mice with a GABA_A_R agonist improved both short and long-term memory formation and memory recall [61]. Importantly, it is apparent there is extensive remodeling of the GABAergic system in AD. Studies have reported region- and cell layer-specific changes to key GABA signaling components in the hippocampus—specifically GABA_A_ receptor subunits and GABA transporters (GATs) responsible for mediating and terminating GABA signaling, respectively [4,23,59,62]. Any functional consequence of these changes has yet to be investigated, but cognition is likely to be affected [62]. As KCC2 and NKCC1 have previously been implicated in altering GABA signaling polarity in neurological disorders such as HD, there is speculation that a similar mechanism is contributing to disease progression in AD [8,63]. The improvement in hippocampal-dependent learning and memory in R6/2 HD mice, following bumetanide treatment, suggests restoring inhibitory GABAergic function in AD may also be therapeutically beneficial [8]. Furthermore, it suggests that as in HD mice, KCC2 and NKCC1 expression may be altered in AD, thus, altering GABA signaling polarity and promoting dysfunction in hippocampal-related learning and memory. Evidence of such alterations has recently been investigated in a mouse model of AD. Mice intrahippocampally injected with Aβ_1–42_ showed upregulation of NKCC1 expression in the CA1 region of the hippocampus [63]. Interestingly, KCC2 was relatively unchanged in the hippocampal CA1 region following Aβ_1–42_ administration. Given the limitations of mouse models such as treatment duration and the inability to fully encapsulate the human disease state, it will be important to expand on this finding in human AD tissue. 

### 4.3. Down Syndrome

DS is a genetic disorder where intellectual disability is a detrimental symptom in children and adults. Individuals with DS have altered hippocampal-related functions that often results in low intelligent quotients, learning deficits, and memory impairment. A mouse model of DS, Ts65Dn, used by Deidda and colleagues [11], is to date the best animal model of DS as these mice carry an extra copy of the mouse chromosome 16 which is syntenic to the human chromosome 21. This DS mouse model replicates characteristic symptoms of the human disease such as hippocampal cognitive deficits involving synaptic plasticity, including long-term potentiation (LTP), as well as learning and memory deficits. Neurodevelopmental alterations observed in Ts65Dn mice include increased generation of forebrain GABAergic interneurons which are thought to be a contributor to the observed impairments [11]. The E/I imbalance in neurotransmission results from excessive inhibition that affects synaptic plasticity and cognition. In this mouse model both cognitive impairments and LTP could be rescued by treatment with GABA_A_R antagonists which reduce the amount of GABAergic signaling [11]. This further enforces the idea that alterations in hippocampal GABA_A_R signaling are linked to LTP abnormalities and cognitive disabilities in DS mice. It is important to note that not all research on DS mice reports enhanced GABA_A_R-mediated inhibition. Whether GABA_A_R signaling is excitatory or inhibitory is also a factor that has developmental implications, and GABA_A_R signaling is excitatory in DS mice. In terms of CCC expression, NKCC1 expression was upregulated in the CA1, CA2, and CA3 regions of the hippocampus, and E_GABA_ was less negative [11]. While the increase in NKCC1 expression was similar to that observed in the human condition, KCC2 expression was largely unchanged in both the DS mouse model and human DS brain tissue. Bumetanide rescued synaptic plasticity and hippocampus-dependent memory in adult Ts65Dn DS mice. Deficits in theta-burst stimulation at Schaffer collateral CA1 synapses, involved in hippocampal LTP, in these mice, were also restored by bumetanide, which suggests the restoration of negative E_GABA_ is crucial for synaptic plasticity. However, while associative, spatial, and recognition memory were restored in Ts65Dn mice, there was no improvement in locomotor activity with bumetanide treatment [11]. This suggests the neurological symptoms of DS may be sufficiently induced by NKCC1 alterations, but the locomotor symptoms may have other underlying mechanisms. 

A study by Parrini and colleagues [64] found similar beneficial effects by using artificial microRNAs (amiRs) to inhibit NKCC1 in the Ts65Dn mouse model. They found RNA interference of NKCC1 restored intracellular Cl^−^ concentrations, GABA-mediated inhibition, and neuronal network dynamics [64]. Mature primary cortical neurons from Ts65Dn mice had reduced intracellular Cl^−^ concentrations which were restored by knocking-down NKCC1 by anti-NKCC1 amiRs. Further cell-attached patch-clamping in mature hippocampal neurons showed bicuculline, a GABA_A_R antagonist, enhanced the average spike frequency in WT cells, indicating GABA was inhibitory. Meanwhile, blocking GABA signaling in Ts65Dn neurons reduced the average spike frequency in 70% of recorded neurons, which suggests GABA is excitatory in these neurons [64]. Endogenous GABA-mediated inhibition was also restored in NKCC1-knockdown Ts65Dn neurons following bicuculline application. This suggests NKCC1-knockdown can rescue altered GABAergic signaling in Ts65Dn neurons. However, spontaneous baseline frequency did not significantly differ between WT and Ts65Dn neurons, which the authors postulated to reflect variability in individual cultured neurons. One interesting finding from this study was the disproportionate neuronal response to GABA—that is, not all neurons responded the same in patch clamp experiments. For example, while the average spike frequency was reduced in 70% of Ts65Dn neurons, the remaining 30% experienced an increase. These findings suggest an increase in the population of neurons which have a depolarizing response to GABA in hippocampal neurons. Immunoblot analysis showed NKCC1 was upregulated in the CA1 region of the hippocampus of Ts65Dn mice, which was reduced by anti-NKCC1 amiRs. However, due to whole lysate analysis using Western blot, this reduction in NKCC1 expression may not be neuronally exclusive and may include other cell types such as glia. Finally, in vivo adeno-associated virus (AAV)-mediated knockdown of neuronal NKCC1 in the hippocampus of Ts65Dn mice improved cognitive deficits in several behavioral tasks such as working memory in the T-maze test, associative memory in the contextual fear conditioning test, and novel object recognition. Long-term effects of in vivo amiR treatment in Ts65Dn mice showed cognitive behaviours were maintained 6 months post-injection, indicating promising results for future therapies [64]. Overall, these findings suggest NKCC1 alterations contribute to behavioral deficits in DS mice and highlight the potential use of gene therapy to treat neurological disorders involving a Cl^−^ imbalance in the CNS. 

### 4.4. Epilepsy

KCC2 and NKCC1 have been implicated in seizure activity as GABA has an essential role in maintaining neuronal function and protecting the CNS against epileptic activity. GABA_A_R activity prevents overexcitation in the CNS by hyperpolarizing postsynaptic membranes and reducing excessive glutamate-mediated excitation, thereby preventing neuronal networks from synchronizing into seizure activity [65]. Hyperpolarization of a neuron involves the influx of Cl^−^ into the cell which is dependent on a low intracellular concentration of Cl^−^ in the cell. A low intracellular Cl^−^ concentration is established by KCC2 which acts as a secondary active transport mechanism by extruding Cl^−^ from the cell in mature neurons [25]. The first 2 weeks of postnatal life in the rat is critical as it is during this period that the excitatory to inhibitory switch in GABA activity occurs. Excessive GABAergic excitation or inhibition can have detrimental effects by enhancing seizure susceptibility or preventing synapse formation, respectively [25,66]. 

Interestingly, GABA_A_R-mediated excitation and spontaneous pyramidal neuron activity have been observed in the subiculum of temporal lobe epilepsy (TLE) patients. Such a finding suggests Cl^−^ is disturbed as both microelectrode recordings and double in situ hybridization revealed low expression of KCC2 in the depolarized subicular pyramidal cells compared with their hyperpolarized counterparts [18]. The seemingly low KCC2 expression in the brains of drug-resistant TLE patients was accompanied by increased NKCC1 expression, and most importantly, a more depolarized E_GABA_ [67,68]. It is reasonable to suspect that altered KCC2 and NKCC1 expression is promoting excitatory GABAergic neurotransmission and disrupting the E/I balance in epilepsy. Furthermore, Sen and colleagues found the upregulation of NKCC1 was neuronally exclusive, despite NKCC1 being expressed both neuronally and non-neuronally, in patients with hippocampal sclerosis—a pathology associated with drug-resistant epilepsy [68,69,70,71,72]. The CA2 region and granule cell layer of the hippocampus are regions which are relatively spared in hippocampal sclerosis, but interestingly, demonstrated increased neuronal expression of NKCC1 [69]. 

As NKCC1 is responsible for Cl^−^ accumulation in immature neurons, a previous study has elucidated that NKCC1 may promote epileptic activity in the developing brain by facilitating the influx of Cl^−^ into hippocampal pyramidal neurons and preventing GABA_A_R-mediated inhibition [69]. The use of bumetanide in epileptic neonatal rats revealed bumetanide reduced cortical seizures, indicating NKCC1 is responsible for epileptiform activity in the developing brain [69]. However, extrapolating these results to the mature brain is questionable as the mechanisms behind epileptic activity in the adult may differ compared with the developing brain. The methodology used by Dzhala and colleagues has also been criticized, as using K^+^ to induce hyperexcitability would undoubtedly affect NKCC1-mediated Cl^−^ transport as it is K^+^-dependent [69]. Most notably, Kilb and colleagues similarly found bumetanide reduced epileptic activity following K^+^-induced hyperexcitability, but could not replicate this finding when using 1 µM kainate to induce hyperexcitability [73]. In fact, inhibiting NKCC1 with bumetanide following kainate-induced excitation increased the frequency of epileptic events in the CA3 region of postnatal day 4–7 mouse hippocampal preparations [73]. Zhu et al. also suggested NKCC1 prevents the development of seizure-like activity, as removing NKCC1 during development, by genetic manipulation or bumetanide application, P9-P13 CA3 mouse pyramidal neurons had increased cellular excitability [66]. Meanwhile, reduced expression of KCC2 promoted seizure susceptibility. These findings are inconsistent with previous literature as Jarolimek and colleagues proposed KCC2 may further exacerbate neuronal hyperactivity by driving an influx of Cl^−^ in response to elevated levels of extracellular K^+^, which drives a hyperactive neuron [74]. Moreover, other studies indicated ubiquitous downregulation of NKCC1 in the developing hippocampus may not occur, and NKCC1 may instead become localized to dendrites as opposed to the soma of interneurons and pyramidal neurons [75]. Thus, the localization of NKCC1 in immature and mature neurons is important to consider when investigating its role in Cl^−^ transport and neuronal excitability. The apparent disparity in epilepsy research regarding the contribution of KCC2 and NKCC1 to neuronal excitability also suggests clarity is needed to fully understand whether applying loop diuretics such as furosemide or bumetanide will be therapeutically beneficial to both neonatal and adult epileptic patients. 

The concept of targeting CCCs as novel anti-epileptic treatments is not new and has been explored in detail [76]. Eftekhari and colleagues demonstrated bumetanide could reduce seizure frequency in adult TLE patients, however given the small sample size, conducting a larger scale study would be encouraged [77]. Kahle et al. had also previously suggested bumetanide reduced seizure duration and frequency in a single case study of a human neonate [24]. More recently, Liu and colleagues found that patients with human focal cortical dysplasia, a common cause of refractory epilepsy, have more internalized KCC2 that is less distributed on the cell membrane in the seizure onset zone of the cortex [78]. Interestingly, this was paired with a more depolarized E_GABA_ and depolarizing GABA activity in pyramidal neurons, which was dampened by bumetanide [78]. However, the small sample sizes of these studies call for a larger scale study to fully elucidate the beneficial effects of bumetanide. Safety issues surrounding the use of diuretics have been waived since furosemide and bumetanide are both FDA-approved loop diuretics and have been widely used for decades. Additionally, while there is no indication of adverse effects following bumetanide treatment, there is a concern around both acute and chronic use of bumetanide on neuronal development and function in neonates [76]. 

There is a similar concern about the use of current antiepileptic drugs (AEDs) for neonatal seizures as administration for long periods, especially during a time of development, is undesirable [79,80]. In addition, around 30% of epileptic patients do not respond to current AEDs so there is a need for novel AEDs [80,81]. Current therapies promote GABA activity but for epileptics without a genetic basis, such as TLE, they may be ineffective. This is because acquired forms of epilepsy from a brain insult, for example, can lead to multiple neurological changes such as neuroinflammation and cell loss, and solely targeting GABA signaling may be insufficient [82,83]. One solution to this therapeutic dilemma may be microRNAs (miRNAs), which have become a target of interest for epilepsy since several miRNA expression profiles appear to be altered in epilepsy; some of which have been linked to functional consequences [82,84,85,86]. MicroRNA are small non-coding RNAs that regulate gene expression. Assembly of miRNAs into RNA-induced silencing complexes (RISCs) can target and recruit mRNAs to degrade or repress them [86,87]. miRNAs such as miRNA-146a are of particular interest as they have the potential to affect several pathways such as neuroinflammation and gliosis, among others [82].

## 5. KCC2 and NKCC1 in Neuropsychiatric Disorders

### 5.1. Schizophrenia

Schizophrenia is a neuropsychiatric disorder that affects 1% of the population and is characterized by a spectrum of symptoms. Symptoms can range from positive ones such as psychosis and hallucinations; negative ones such as social withdrawal and loss of motivation and energy; and cognitive symptoms such as working memory deficiencies [88]. 

As the gross pathology of schizophrenic brains is not well established, it is suggested that observing specific cell populations and their functionality, and cell–cell communications, is better to characterize the disorder [89]. On the molecular level, pharmacological studies have insinuated neurotransmitter abnormalities, but this is difficult to relate to the clinical symptoms. Thus, genetic risk factors have emerged as the most effective way of identifying disease markers, particularly genes associated with the underlying disease mechanisms [89]. 

Post-mortem studies have demonstrated several NT systems are altered in schizophrenia, but it is criticized as to whether such alterations contribute to the primary pathogenesis [89]. The loss of glutamatergic neurons, among other abnormalities, in the post-mortem schizophrenic tissue is also variable and inconsistent between studies [89,90]. Conversely, GABAergic abnormalities in post-mortem tissue have been more consistent with downregulation of *GAD1*, which encodes the enzyme responsible for GABA synthesis, and altered molecular markers indicating reduced GABA activity [19,88,91]. Of particular interest are GABAergic parvalbumin (PV) neurons within the prefrontal cortex of the brain which can produce abnormal gamma frequency oscillations and affect cortical activity, specifically working memory function [91]. 

Several risk-associated genes are preferentially expressed in development which suggests that neuronal development is a key player in developing schizophrenia [47,92,93,94,95]. For example, neuregulin 1 (*NRG1*), encodes the NRG1 trophic factor involved in neural development, which regulates GABA receptor and interneuron expression and activity by stimulating GABA release from interneurons and inhibiting pyramidal neurons of the prefrontal cortex [92]. Mice knock-out studies of the NRG1 receptor, ErbB4, in PV-interneurons produced schizophrenic phenotypes such as impaired working memory and hyperactivity [92].

It is uncertain whether GABAergic alterations are a secondary result of the primary disease mechanisms involving reduced glutamatergic activity. However, GABAergic interneurons differentiate into diverse populations with different functions throughout development. The diversity of neurons in the GABAergic system and their variety of neuronal connections showcase how necessary it is for interneurons to have coordinated development and function in several brain regions, so it is plausible that glutamatergic dysfunction begins with altered GABA activity [88]. KCC2 and NKCC1 dysregulation have been reported in schizophrenia using real-time quantitative polymerase chain reaction (RT-qPCR) in the prefrontal cortex, which may affect the E/I switch in the affected individuals [96]. Hyde and colleagues [19] also reported increased GAD25/GAD67 and NKCC1/KCC2 ratios in schizophrenic patients. Both GAD25 and NKCC1 are preferentially expressed early in development and are involved in GABA synthesis and depolarizing GABA activity, respectively, thus, implying an immature GABA physiology in schizophrenia. Furthermore, the ratios are associated with the rs3749034 genotype which is a risk-associated promoter SNP in *GAD1*, an enzyme involved in GABA synthesis, overall suggesting abnormal GABA signaling in development is a genetic risk for schizophrenia [19]. Post-mortem studies of schizophrenic patients show *SLC12A5*, the gene which encodes KCC2, is reduced in the hippocampus [97], whereas, schizophrenic patients have shown an upregulation of the NKCC1 gene, *SLC12A2*, in the prefrontal cortex [95]. A gain-of-function variant discovered in genetic studies also demonstrates NKCC1 activity may be activated where it would normally be silent in schizophrenic patients [98]. A study by Callicott et al. found NKCC1 could regulate hippocampal neuron development by interacting with the gene dissociated in schizophrenia 1 (DISC1), which may be altered in schizophrenic patients [47]. Wang and Kriegstein explored the use of bumetanide in immature mouse neurons and notably found that by blocking NKCC1 with bumetanide, these neurons significantly reduced AMPA receptor-mediated depolarizing synaptic currents once mature [48]. Thus, NKCC1 is necessary for the development and formation of excitatory synapses in neurons. 

It is important to note that studies suggest genetic susceptibilities are more likely to manifest as a schizophrenic disorder when combined with environmental insults such as maternal depression, advanced paternal age, or perinatal hypoxia, among others [88]. The GABAergic system is a likely candidate for dysregulation following an environmental insult as it plays both excitatory and inhibitory roles depending on the stage of development. For example, DISC1 regulation of neuronal development and hippocampal neurogenesis is dependent on GABA-mediated excitation, and downregulation of DISC1 can lead to enhanced neuronal integration and aberrant morphological development [93,94]. Acceleration of the E/I switch in GABA activity can also lead to failure of DISC1-dependent neuronal development [93]. The impact of altered CCC expression on schizophrenic behaviors is not well understood and requires further research. Given the diversity of the GABAergic system, it is also difficult to pinpoint which interneuron population is affected. Multiple interneuron populations have been implicated in schizophrenia, such as prefrontal PV-interneurons which demonstrate downregulation of GAD67 and GAT1, indicating the deficits are occurring at the level of neuronal networks [91]. 

### 5.2. Autism Spectrum Disorders

ASDs are developmental disorders associated with communication difficulties, social disabilities, and repetitive behaviors. Several animal models of autism exhibit E/I imbalances. Gamma frequency oscillations, involved in cognitive function, are decreased in autism [99]. While the mechanisms underlying ASDs are unknown, it is suggested that alterations in neuronal development, including an E/I imbalance, can affect development of emotional, memory, and sensory systems [100,101]. Although ASDs cover a broad range of disorders, such as Rett syndrome and FRX, they have overlapping symptoms, thus, are likely to share the same mechanisms behind the neurodevelopmental deficits [102]. 

FRX is associated with mutations in the *FRM1* gene, which encodes the *Fragile X* mental retardation protein (FMRP), on chromosome X, mutations of which result in the FMRP not being expressed and an intellectually disabled phenotype. In Fmr1 KO mice, there is an increase in network excitability and an E/I imbalance [102,103,104]. In rodents, the delivery is critical to the excitatory-to-inhibitory switch in GABA signaling as the release of oxytocin reduces [Cl^−^]*_i_* and exerts analgesic and neuroprotective effects. Studies found the transition of GABA from excitatory to inhibitory is associated with the delivery process in rodents as it was found that the transition was abolished in a drug-induced and FRX rodent model of autism. Furthermore, they found depolarizing GABA activity was due to decreased KCC2 expression and subsequent high [Cl^−^]*_i_* [105]. 

The X-linked *Mecp2* gene encodes MeCP2 which has high expression in GABAergic interneurons. In RTT, mutations in the *Mecp2* gene result in symptoms such as loss of speech, respiratory dysrhythmias, and cognitive deficits. Similar to FRX, a reduction in KCC2 and an increase in NKCC1 have been reported in RTT [102,106]. Studies also found a significant reduction in cerebrospinal fluid KCC2 in RTT and a reduced KCC2-to-NKCC1 ratio, suggesting GABAergic neuronal maturation is disturbed in the disorder.

GABAergic dysfunction in ASDs is well-studied, however, the use of GABA_A_R allosteric modulators such as benzodiazepines has been associated with increasing aggression and anxiety [102,107]. Thus, alternatives to allosteric modulators include the NKCC1 inhibitor, bumetanide. Clinical trials have shown that bumetanide can reduce autism severity with little adverse events [99,108,109]. The long-term treatment of bumetanide was tested in a clinical trial in children with autism or Asperger syndrome where they received 1 mg daily of bumetanide, following a promising pilot study [99,109]. Side effects of an electrolyte imbalance following bumetanide administration were noted, where mild hypokalemia was observed in 30% of patients, and treated with supplemental potassium, thus, the authors concluded that bumetanide may be safe for long-term administration with minimal side effects [99]. However, one child was removed from the study for their hypokalemia, suggesting a potential issue involving this side effect. A following study found bumetanide can improve ASD symptoms with a minimal risk at a dose of 1 mg twice daily [108]. Chronic bumetanide treatment improves emotion recognition and enhances the activity of brain regions involved in emotional and social perception [110]. Thus, bumetanide may be useful in improving social processing in autism.

## 6. Pharmacological Targeting of the Cation-Chloride Cotransporters

### 6.1. NKCC1 as a Therapeutic Target

**Figure 1:**
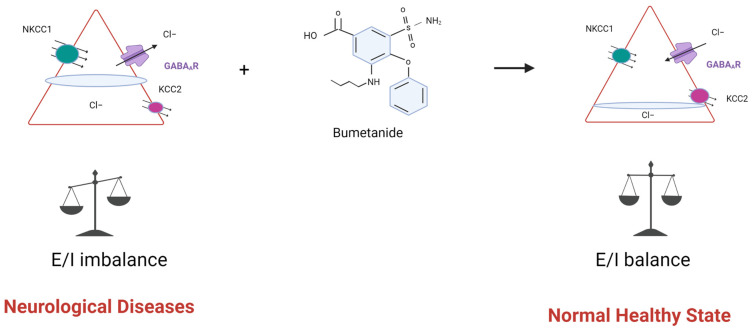
Dysregulation of chloride homeostasis leads to excitatory/inhibitory (E/I) imbalance. An altered expression of KCC2 and NKCC1 in neurological diseases including Alzheimer’s, Huntington’s, and Parkinson’s disease, schizophrenia, Down syndrome and epilepsy could disrupt GABAergic transmission and promote an E/I imbalance. GABA_A_ receptor (GABA_A_R) activation can shift the response from hyperpolarizing to depolarizing, contributing to enhanced network excitability. This process can be returned to normal by blocking NKCC1 with bumetanide.

Pharmacologically, CCCs are targeted by loop diuretics such as bumetanide and furosemide [37]. Bumetanide is a FDA-approved loop diuretic that acts as an NKCC1 antagonist to treat high blood pressure and other conditions [111]. As NKCC1 has been linked to promoting excitatory GABAergic activity, NKCC1 inhibitors and KCC2 activators are being evaluated as potential therapies for neurological and neuropsychiatric disorders. A recent discussion arose around the repurposing of bumetanide to treat AD as in the race to find the cure to AD an old FDA-approved drug would, in theory, accelerate the extensive process of clinical trials [112,113,114]. Several neurological, neurodevelopmental, and psychiatric disorders have been linked to dysregulation of the excitatory-to-inhibitory switch in GABAergic neurotransmission, thus, restoring the ionic balance in GABAergic neurons and GABAergic inhibition may be of therapeutic benefit to these disorders [20] (Figure 1). Current evidence surrounding such neurological disorders often points to an upregulation of NKCC1 and downregulation of KCC2 expression in the adult state. This creates a contradiction in the normal expression of KCC2 and NKCC1, as typically KCC2 is more highly expressed than NKCC1 in mature neurons. Consequently, the altered expression of KCC2 and NKCC1 could promote an E/I imbalance and disrupt GABAergic transmission [25]. An increase in NKCC1 expression may increase intracellular Cl^−^ concentrations, thus, altering GABA-mediated inhibition and enhancing neuronal excitation (Figure 1). Bumetanide had previously shown neuroprotective effects in both in vitro and in vivo models of cerebral ischemia whereby the drug reduced infarct volume by 25% following a middle cerebral artery occlusion [115,116]. The mechanism may involve the prevention of increased intracellular Cl^−^ during reoxygenation or reducing the NKCC1-mediated Na^+^ influx and preventing excitotoxicity. 

Most recently, Taubes and colleagues explored the use of bumetanide as a drug candidate for apolipoprotein ε4 (APOE4)-related AD [114]. Bumetanide has the neuroprotective potential to rescue neurons from Aβ_1–42_-induced excitotoxicity by re-establishing the E/I balance in AD, as observed in cerebral ischemia models [63,115,116]. The improved hippocampal-dependent memory and synaptic plasticity in DS mouse models and abolishment of neuronal excitability and restoration of inhibitory drive in HD mice are also a testament to the therapeutic potential of bumetanide in AD [8,11] (Table 1). The challenge with testing drug candidates for neurological disorders is maintaining efficacy in human trials. Human studies are the gold standard for evaluating whether a drug is suitable, but they do not have the same homogeneity and reliability that animal studies have, leading to unprecedented results. However, the one-size-fits-all approach rarely works in most instances. Taubes et al. have suggested a personalized medicine approach towards disorders such as AD where the genetics, pathology, and clinical appearance and symptom onset differ between individuals [114]. The *APOE4* genotype represents the largest genetic risk factor for late-onset AD and an ideal subpopulation for drug development [23]. Taubes and colleagues used bumetanide to alter the expression of the ε4 allele of apolipoprotein E (APOE4), the strongest genetic risk factor for sporadic AD [114]. Extensive investigation was undertaken to identify any AD gene expression signature that could be driven by APOE4. The candidate E4 for AD was chosen, and the search was then expanded to look for approved drugs that could reverse or flip E4 to a more regular E3 state. Using a drug-repurposing algorithm, bumetanide was identified as the highest-ranking drug for *APOE4/APOE4* AD, one of the most efficacious in reversing the transcriptomic signature of brain aging in *APOE4*-knock-in (KI) mice and was capable of rescuing LTP deficits in older *APOE4*-KI mice, which affects neuronal plasticity and memory formation [117]. Previously, work had been conducted on only identifying molecules that alter molecular signatures in cells that carry AD risk genes. It was found that bumetanide reversed the signatures of cultured human excitatory, inhibitory and dopaminergic neurons derived from APOE4/4 induced pluripotent stem cells. As well as in vitro measures, Taubes and colleagues treated 16-month-old APOE4/4 knock-in mice with 0.2 mg/kg of bumetanide for eight weeks, and found the RNA-sequence for APOE4 transcriptome had flipped in various cell types and not just in neurons [114].

Based on these findings, the methods used by Taubes and colleagues may be extended to several neurological disorders to identify suitable drug candidates [114]. The field of drug discovery and development is fundamental to initiating in vitro and in vivo experiments and clinical trials. Any potentially unfit drug candidates in the vast drug database can be filtered out computationally to avoid clinical issues such as drug–drug interactions and affinity to the target site. This promising approach to drug discovery has however, been challenged by a realistic pharmacological outcome. Bumetanide poorly penetrates the brain and may have issues reaching the target site. It is also a non-selective inhibitor of NKCC1 and may cause excessive diuresis by inhibiting NKCC2 in the kidney [113]. Most recently, Savardi and colleagues used an integrated drug discovery strategy to identify novel candidates to inhibit NKCC1 [120]. Two novel drug candidates, ARN22642 and ARN22430, potently inhibited NKCC1 with low in vitro solubility and metabolic stability. Multiple assays showed high selectivity for NKCC1 for both drug candidates. When tested in the Ts65Dn DS mouse model, ARN23746, an analog of ARN22430 with improved potency and NKCC1 selectivity, reduced intracellular Cl^−^ to physiological levels in DS mouse neurons. In addition, ARN23746 had an improved pharmacokinetic profile in vivo, rescued cognitive deficits in Ts65Dn mice, and had no significant diuretic effect in wild-type or DS mice compared with bumetanide [120] (Table 1). However, Raveendran and colleagues gave a word of caution with selectively targeting NKCC1, as it is ubiquitously expressed, and chronic treatment with NKCC1 inhibitors such as bumetanide can lead to ototoxicity in the inner ear [126,127,128]. A recent study also suggested bumetanide is neurotoxic even at low micromolar concentrations as primary hippocampal mouse neurons had greater cell death with bumetanide than Aβ_1–42_ alone [63]. Thus, NKCC1, although lowly expressed in mature neurons, is essential for normal physiological function, and treatment with bumetanide may be detrimental to normal NKCC1 activity. It is possible that bumetanide prompted an ionic imbalance in these neurons and by impairing the NKCC1-mediated influx of Cl^−^ and promoting Cl^−^ efflux via KCC2 and GABA_A_R-mediated hyperpolarization [63]. It is possible that primary mouse hippocampal neurons show high sensitivity to the drug and these adverse effects might not affect hippocampal neurons in vivo. Furthermore, the concentration of bumetanide might not reach such high concentrations in the brain in vivo. Therefore, while the in vitro neurotoxicity of the drug is concerning, its effect on the brain must be further explored in in vivo experiments. Bie and colleagues found bumetanide improved cognitive performance in the Morris water maze test in a rat AD model with reduced KCC2 expression, indicating bumetanide may not be neurotoxic in vivo [118]. Another study by Flagella et al. demonstrated that inhibiting NKCC1 in NKCC1-KO mice disrupts the K^+^ influx and secretion into the endolymph, resulting in balance impairments and deafness [129]. In the perfused rat liver, bumetanide blocked the volume-regulatory net K^+^ uptake which promoted cell shrinkage and potentially the generation of reactive oxygen intermediates and oxidative stress, which would likely be detrimental to cell survival [130]. It was also postulated that the bumetanide-induced neuronal cell death is non-specific, as Pond and colleagues reported that bumetanide at higher concentrations has less neuroprotective capabilities and more non-specific toxic effects following oxygen–glucose deprivation [115]. In this study, the maximum effective concentration of bumetanide protected CA1 neurons but also had the potential to inhibit KCC2, which would promote cell death and swelling [115,131]. Indeed, the suitability of bumetanide as therapy in diseases involving altered KCC2 or NKCC1 expression is still questionable, but worth investigating. 

Recently, a study investigated the clinical efficacy of bumetanide in young ASD patients. Bumetanide was administered twice daily at a dose of 0.5 mg and reduced symptom severity based on the Children Autism Rating Scale (CARS) score [132]. The study also investigated the GABA-to-glutamate ratio in the insular and visual cortices—regions associated with managing cognition and behavior in response to emotional, sensory, and autonomic stimuli. They found a reduction in the GABA-to-glutamate ratio in the insular and visual cortices, which was associated with improved clinical symptoms [132]. Previous mouse models of ASD found enhancing GABAergic inhibition rescued impaired sensory integration in the insular cortex [29]. Bumetanide also reduced hallucinations in adolescent and adult schizophrenic patients, an effect that continued during a one-month wash out period [124,125]. 

Of relevance, repurposing bumetanide has extended to PD, a disorder characterized by uncontrollable motor symptoms, such as a resting tremor. Importantly, bumetanide improved PD motor symptoms in a clinical trial with four patients [17,113] and in a rat model of PD [119]. Increased levels of Cl^−^ are thought to contribute to motor symptoms caused by dopamine deficiency. Thus, reducing intracellular Cl^−^ using bumetanide may ameliorate these symptoms. Since bumetanide was well-tolerated in the small pilot study conducted on four PD patients, a phase II clinical trial was initiated on a larger cohort of 40 PD patients [17,113]. However, the results of this clinical trial have yet to be released. 

### 6.2. KCC2 as a Therapeutic Target

KCC2 stimulators have also been explored as a therapeutic alternative as KCC2 is dominantly expressed in mature neurons [25] (Table 1). As for NKCC1, KCC2 is involved in normal physiological function, and targeting a naturally expressed protein may have detrimental effects. Unrelated to regulation of GABAergic neuronal activity, KCC2 is involved in cell volume regulation by promoting K^+^, Cl^−^ efflux, and water efflux. In isotonic conditions, KCC2 maintains K-Cl cotransport due to its unique C-terminal domain [133,134]. 

Treatments for neonatal seizures have focused on targeting NKCC1, but an alternative could be to enhance KCC2 activity. KCC2 is not as ubiquitously expressed as NKCC1 as it is localized to neurons, thus, would be less risky in terms of systemic side effects [70,135]. Lack of efficacy of the first-line therapy for pediatric seizures, phenobarbital, was postulated to be attributed to reduced abundance and function of KCC2 by Sullivan and colleagues [121]. Their study found that in a CD-1 mouse model of refractory ischemic neonatal seizures, CLP290, a KCC2 enhancer, reduced seizure frequency and duration [121]. Thus, systematic administration of CLP290 can restore phenobarbital efficacy and KCC2 deficits could be causal to phenobarbital resistance. Importantly, the efficacy of CLP290 was dependent on KCC2 phosphorylation as the phosphorylation status of KCC2 affects its function. For example, phosphorylation of serine 940 by protein kinase C (PKC) slows down transporter endocytosis and increases KCC2 activity, whereas phosphorylation of threonines 906 and 1007 by WNK kinase decreases the intrinsic rate of KCC2 transport [136]. Thus, the phosphorylation status of KCC2 should be considered when developing new therapies. 

The use of KCC2 stimulators has also been investigated in spinal cord injury. The KCC2 activator, CLP257 was developed following a high-throughput screening of drug-like compounds by Gagnon and colleagues [123]. CLP257 is selective for KCC2 compared with other KCCs and can reduce intracellular Cl^−^ by enhancing KCC2-mediated Cl^−^ extrusion. In a rat model of neuropathic pain, CLP257 enhanced Cl^−^ extrusion by increasing KCC2 cell surface expression. Such models use brain-derived neurotrophic factor (BDNF) to downregulate KCC2 and mimic pain hypersensitivity. However, the involvement of BDNF in KCC2 expression is complex as BDNF has a wide spectrum of effects on neuronal maturation. It is likely that neuronal interactions, among other factors, affect KCC2 expression [137]. Furthermore, in lieu of the promising therapeutic benefit presented by CLP257, the compound had poor pharmacokinetic properties such as a considerably low plasma half-life. Consequently, CLP290 was developed as a carbamate prodrug with an improved plasma half-life, oral efficacy, and blood–brain barrier penetrability [123]. CLP290 did not share the sedative and dizziness side effects associated with pregabalin, an anticonvulsant drug used for neuropathic pain. Thus, CLP290 appeared to be well tolerated. CLP290 has been optimized for systemic administration [123] and animal models have shown that CLP290 is effective in treating neuropathic pain, a common complication following spinal cord injury, compared with other compounds. CLP290 also demonstrated minimal side effects when administered at high doses [122]. In addition, a study by Chen et al. investigated CLP290 as a therapy for spinal cord injury and found the KCC2 agonist restored the stepping ability of paralyzed mice with spinal cord injury [122]. Spinal cord injury can lead to complete paralysis as remaining axons fail to facilitate a functional recovery [122]. Bumetanide, on the other hand, had no significant effect. It appeared that enhancing KCC2 expression in inhibitory interneurons allowed for improvements towards functional recovery following spinal cord injury. Additionally, altered expression of KCC2 and NKCC1 following spinal cord injury may contribute to the development of neuropathic pain. For more information, the role of CCCs in spinal cord injury and neuropathic pain was recently reviewed in detail by Talifu and colleagues [138].

Recently, Zhang and colleagues developed 5-chloro-N-(5-chloro-4-((4-chlorophenyl)(cyano)methyl)-2-methylphenyl)-2-hydroxybenzamide (ZT-1a), a potent and selective inhibitor of SPS1-related proline/alanine-rich kinase (SPAK) [100]. SPAK, encoded by the STK39 gene, is a CCC regulator that stimulates NKCC1 and inhibits KCC activity. Increased SPAK-dependent phosphorylation of CCCs has been postulated to be involved in neurological disorders. ZT-1a hinders SPAK-dependent phosphorylation of CCCs, thus, inhibiting NKCC1 and stimulating KCC activity. In ischemia and other brain injuries, subsequent impaired cell volume regulation can result in cell swelling, cerebral oedema, and disrupted integrity of the blood-brain barrier [139]. The study by Zhang et al. found ZT-1a reduced ischemia-induced phosphorylation of CCCs, protected against ischemic brain damage, and attenuated cerebral edema [100]. Thus, ZT-1a may be a promising treatment for dysregulations in brain volume homeostasis. However, the study broadly focused on KCCs with particular emphasis on KCC3, which is also abundantly expressed outside the CNS. It would be worth investigating the effect of ZT-1a on KCC2 activity specifically in neurons. In addition, the authors noted the pharmacokinetic profile of ZT-1a can be improved as its plasma half-life is less than 2 h and it minimally penetrates the blood–brain barrier [100]. Four derivatives of ZT-1a (ZT-1c, -1d, -1g, and -1h) have since been developed, but their efficacies have yet to be investigated in vivo [140].

## 7. Conclusions

The CCCs, KCC2 and NKCC1, may provide a promising new target for neurological and neuropsychiatric disorders such as AD, HD, PD, DS, epilepsy, schizophrenia, and ASDs. While several other neurological disorders and insults such as cerebral edema, spinal cord injury, and neuropathic pain have not been covered in this review in detail, the research on the disorders mentioned here provides some insight into how bumetanide or other CCC targeting therapies could benefit a variety of disorders [20]. The role of CCCs in other neuropsychiatric disorders such as bipolar disorder, depression, or attention deficit hyperactivity disorder (ADHD) have yet to be explored, thus, this review focused on schizophrenia and autism. GABAergic dysfunction underlies many neurological abnormalities and has recently been a target of interest as inhibiting NKCC1 with bumetanide could attenuate the E/I imbalance in these disorders [23,141]. While bumetanide is a promising therapy for neurological disorders involving an E/I imbalance, this approach needs to be refined to enhance brain penetration and selectively target NKCC1 to enhance efficacy and reduce potential peripheral side effects [113]. In addition, the mechanisms involved in dysregulation of KCC2 and NKCC1 activity or expression may differ between brain disorders. Drugs with differing pharmacokinetic properties need to be examined to better tackle each brain disorder in this case, and selective targeting of specific neuronal subpopulations must be considered as well. However, overall, the GABAergic system and the CCCs remain promising targets for new therapies. 

## Figures and Tables

**Table 1:** Summary of CCC alterations in neurological and neuropsychiatric disorders, and the therapeutic effect of bumetanide and other CCC targeting therapies in these conditions. Bumetanide = NKCC1 antagonist; ARN23746 = NKCC1 antagonist; amiRs = artificial microRNAs; CLP290 = KCC2 activator; CLP257 = KCC2 activator; ZT-1a = selective SPAK inhibitor. TLE = temporal lobe epilepsy; FCD = focal cortical dysplasia; SCI = spinal cord injury; VPA = valproate; RTT = Rett syndrome; FRX = Fragile X mutation; ASD = autism spectrum disorder.

	Authors	Study Design	Drug	CCC Expression	Outcomes
**Neurological Disorders**					
Huntington’s disease	Dargaei et al. [8]	R6/2 and YAC128 HD mice	Bumetanide	Increased NKCC1 protein. Decreased KCC2 protein (oligomer + monomer); decreased KCC2 fluorescence at surface membrane of hippocampus.	Improved behavior, learning, and memory; restored E_GABA._
Alzheimer’s disease	Lam et al. [63]	Mouse hippocampal primary neurons AD mouse model	Bumetanide -	No change. Increased NKCC1 fluorescence and density. Decreased KCC2 fluorescence and density.	Neurotoxicity at concentrations 1 µM, 10 µM, 100 µM, and 1 mM. -
	Bie et al. [118]	Rat AD model	Bumetanide	Decreased KCC2 protein.	Improved cognition and normalized GABAergic E_Cl−._
Parkinson’s disease	Damier et al. [17]	PD patients	Bumetanide	-	Improved motor symptoms.
	Lozovaya et al. [119]	Rat PD model	Bumetanide	-	Improved motor symptoms.
Down syndrome	Deidda et al. [11]	Ts65Dn mouse model DS patients	Bumetanide -	Increased NKCC1 protein and surface expression. Increased NKCC1 protein and mRNA.	Rescued associative, spatial and recognition memory, but not locomotor activity.
	Savardi et al. [120]	Ts65Dn mouse model	ARN23746	-	Restored [Cl^−^]*_i_* and rescued cognitive deficits.
	Parrini et al. [64]	Ts65Dn mouse model	amiRs	Increased NKCC1 protein.	Restored [Cl^−^]*_i_* and GABAergic inhibition.
Epilepsy	Dzhala et al. [69]	Epileptic neonatal rats	Bumetanide	-	Reduced cortical seizures.
	Kilb et al. [73]	Mouse hippocampal neurons	Bumetanide	-	Reduced epileptic activity.
	Zhu et al. [65]	Mouse hippocampal neurons	Bumetanide	Decreased KCC2 protein.	Increased cellular excitability.
	Eftekhari et al. [77]	Adult TLE patients	Bumetanide	-	Reduced seizure frequency.
	Kahle et al. [24]	Human neonate	Bumetanide	-	Reduced seizure frequency
	Liu et al. [78]	FCD patients	Bumetanide	Decreased KCC2 protein and surface expression; increased KCC2 internalization.	Reduced depolarizing GABA activity.
	Sullivan et al. [121]	CD-1 mouse model	CLP290	-	Reduced seizure frequency and duration.
	Palma et al. [67]	TLE patients	Bumetanide	Increased NKCC1 mRNA and decreased KCC2 mRNA in hippocampal subiculum	More negative shift in E_GABA_.
Spinal Cord Injury	Chen et al. [122]	SCI mouse model	CLP290	-	Restored stepping ability in paralyzed mice with SCI.
			Bumetanide	-	No significant effect.
Neuropathic Pain	Gagnon et al. [123]	Rat spine slices with peripheral nerve injury	CLP257	Reduced KCC2 surface protein.	Restored Cl^−^ transport, hyperpolarized E_GABA_, and increased KCC2 cell surface expression.
Ischemia	Zhang et al. [100]	Mouse model of ischemic stroke	ZT-1a	-	Blocked ischaemia-induced phosphorylation of CCCs; reduced NKCC1 and increased KCC3 activity. Reduced infarct volume and ischemic cerebral oedema; improved neurological function; protected gray and white matter tissue.
	Yan et al. [116]	Rat focal ischemia model	Bumetanide	Increased total and phosphorylated NKCC1 protein in the cortex.	Reduced infarct volume.
**Neuropsychiatric Disorders**					
Schizophrenia	Hyde et al. [19]	Schizophrenic patients	-	Increased NKCC1-to-KCC2 mRNA ratio; decreased KCC2 mRNA in hippocampus.	-
	Tao et al. [97]	Schizophrenic patients	-	Decreased *SLC12A5* gene in hippocampus.	-
	Dean et al. [95]	Schizophrenic patients	-	Increased *SLC12A2* gene in prefrontal cortex.	-
	Lemonnier et al. [124]	Adolescent schizophrenic patients	Bumetanide	-	Reduced hallucinations.
	Rahmanzadeh et al. [125]	Schizophrenic patients	Bumetanide	-	Reduced refractory hallucinations.
Autism	Tyzio et al. [105]	VPA rats and FRX mice	-	Decreased KCC2 protein; shift in KCC2 fluorescence labeling from cell membrane to cytoplasm.	-
	Duarte et al. [106]	RTT patients	-	Decreased KCC2 protein in CSF; decreased KCC2-to-NKCC1 protein ratio.	-
	Lemonnier et al. [108]	Children with ASD	Bumetanide	-	Improved ASD symptoms.
	Hadjikhani et al. [110]	Adolescents and young adults with autism	Bumetanide	-	Improved emotion recognition; activated brain regions involved in social emotional and social perception.

## Data Availability

Not applicable.
